# Act together—implications of symbioses in aquatic ciliates

**DOI:** 10.3389/fmicb.2012.00288

**Published:** 2012-08-07

**Authors:** Claudia Dziallas, Martin Allgaier, Michael T. Monaghan, Hans-Peter Grossart

**Affiliations:** ^1^Marine Biological Section, University of CopenhagenHelsingør, Denmark; ^2^Department of Limnology of Stratified Lakes, Leibniz-Institute of Freshwater Ecology and Inland FisheriesStechlin, Germany; ^3^Berlin Center for Genomics in Biodiversity ResearchBerlin, Germany; ^4^Department of Limnology of Shallow Lakes and Lowland Rivers, Leibniz-Institute of Freshwater Ecology and Inland FisheriesBerlin, Germany; ^5^Institute for Biochemistry and Biology, Potsdam UniversityPotsdam, Germany

**Keywords:** aquatic ciliates, ciliate-bacteria interaction, symbiosis, associated prokaryotes, microbial functions, ecosystem function

## Abstract

Mutual interactions in the form of symbioses can increase the fitness of organisms and provide them with the capacity to occupy new ecological niches. The formation of obligate symbioses allows for rapid evolution of new life forms including multitrophic consortia. Microbes are important components of many known endosymbioses and their short generation times and strong potential for genetic exchange may be important drivers of speciation. Hosts provide endo- and ectosymbionts with stable, nutrient-rich environments, and protection from grazers. This is of particular importance in aquatic ecosystems, which are often highly variable, harsh, and nutrient-deficient habitats. It is therefore not surprising that symbioses are widespread in both marine and freshwater environments. Symbioses in aquatic ciliates are good model systems for exploring symbiont-host interactions. Many ciliate species are globally distributed and have been intensively studied in the context of plastid evolution. Their relatively large cell size offers an ideal habitat for numerous microorganisms with different functional traits including commensalism and parasitism. Phagocytosis facilitates the formation of symbiotic relationships, particularly since some ingested microorganisms can escape the digestion. For example, photoautotrophic algae and methanogens represent endosymbionts that greatly extend the biogeochemical functions of their hosts. Consequently, symbiotic relationships between protists and prokaryotes are widespread and often result in new ecological functions of the symbiotic communities. This enables ciliates to thrive under a wide range of environmental conditions including ultraoligotrophic or anoxic habitats. We summarize the current understanding of this exciting research topic to identify the many areas in which knowledge is lacking and to stimulate future research by providing an overview on new methodologies and by formulating a number of emerging questions in this field.

## Introduction

Ciliates are an extraordinarily widespread group of protists that occur in almost all aquatic environments. These include coastal waters, hydrothermal vents, anoxic sediments, hyporheic zones, and oxic as well as anoxic parts of the water column. Ciliates are very abundant phagotrophs in the biosphere and are capable of forming extensive blooms. They are important grazers of algae, bacteria, and other microorganisms (Taylor and Sullivan, 1984; Sherr and Sherr, 1987; Eppstein, 1997). They promote the re-mineralization of microbial biomass and increase the transfer of nutrients to other organisms (Vickerman, 1992). The global diversity of free-living ciliates is surprisingly low (ca. 3000; Finlay et al., 1998), but this number has been called into question. Foissner and colleagues (2007) calculated that there are between 27,000 and 40,000 free-living ciliate species by using the moderate endemicity model. An estimation of ciliate species diversity based on small-subunit ribosomal sequences seems to be unreliable (Nebel et al., 2011) due to the fact that sequence similarity among different species can vary widely (see Caron et al., 2012).

Endosymbiosis may represent a general evolutionary strategy by which phagotrophic protists acquire novel metabolic functions such as photosynthesis, nitrogen fixation and recycling, methanogenesis, and sulphide oxidation. They can therefore be regarded as an important source of genetic innovation (Nowack and Melkonian, 2010). Protists harbor bacteria, algae, fungi, and viruses (Gibson, 1974) and are regarded as popular symbioses initiators with other microorganisms, particularly bacteria (Soldo, 1987). Such symbioses may be more widespread among protists than previously thought (Gast et al., 2009). In ciliates, both endosymbionts and ectosymbionts have been repeatedly reported in aerobic and anaerobic environments (Rosati, 2004). It is thought that ciliates had a photosynthetic ancestor (Reyes-Prieto et al., 2008) but lost their plastids. This would decrease the number of required symbioses for the development of plastids (Archibald, 2008). Today, ciliates host plastids as well as other ecto- and endosymbionts, a fact known since the late 19th century.

Despite their early discovery, the diversity and ecological function of ciliate symbioses are surprisingly little understood. Here we attempt to summarize symbiotic interactions with ciliates and their ecological implications for both freshwater and marine ecosystems. We also highlight a number of emerging research questions regarding the dynamics and ecological traits of symbioses between the ciliate host and its prokaryotic symbionts. We maintain that these model organisms may enlighten more general processes of establishment and maintenance of symbiosis.

## An overview of symbioses

We use here symbiosis sensu De Bary (1879) who defined it as long-term interactions between two different organisms. Symbionts may affect their hosts in a positive or negative manner (Table 1). They have the potential to significantly affect the ecology, physiology, and evolution of both partners (Cavanaugh et al., 2006; Gast et al., 2009) and may therefore facilitate the occupation of new ecological niches and have an impact on whole food webs and ecosystems (Görtz and Brigge, 1998; Table 2). Symbionts and hosts represent consortia with two or more coexisting and interacting genomes. These form metabolic competencies and natural selection therefore operates on these integrated consortia (Finlay, 2004).

**Table 1:** **Effects of symbiosis on ciliate hosts and symbionts**.

	**Effects on host**	**Effects on symbiont**
Positive	Supply of nutrients and organic matter, growth factors, vitamins etc. Competitive advantage Protection against parasites Oxygen removal by heterotrophs Degradation of metabolic waste; detoxification Protection against UV radiation Adaptation of aerobic life in anoxic zones	Nutrient supply movement to favorite conditions, increased motility grazing protection disposal of organic or inorganic material Less competitors Supply with CO_2_ and H_2_
Neutral or unclear	Space requirements by symbionts H_2_ scavengers in anaerobic ciliates (backup for methanogens?) Energy transfer? Higher grazing pressure?	Better genetic exchange? Constant conditions, e.g., pH?
negative	Competition for nutrients and organic matter cell lysis inhibition by toxins etc.	genetic bottleneck effect digestion by the host

**Table 2:** **Potential and *published* ecological consequences of symbioses in aquatic ciliates (for references see text)**.

	**Effects of the entity host-symbiont**
Material cycling	***High photosynthesis rates*** ***Methanogenesis*** ***Sulfur transformation*** ***Demands on iron and magnesium*** Nitrogen fixation Phosphate storage
Other organisms	***Competitive advantage*** ***Toxin production*** Reservoir and vector for pathogens
Ecosystem	***Covering new ecological niches*** Higher autochthon biomass formation Strengthening of the microbial loop

Symbioses can be mutualistic, commensalistic, or parasitic. Most symbioses investigated to date are mutualistic. Common examples include symbioses with photoautotrophs, lithoautotrophic prokaryotes, and organoheterotrophic bacteria. Mutualism benefits both partners by nutrient and energy supply, and through protection from predators or environmental threats such as oxygen radicals and toxins (Boucher et al., 1982). Many mutualistic microbial symbioses have a biochemical origin, based on the transfer of compounds produced by one partner or the other (Hoffmeister and Martin, 2003). Mutualistic symbioses increase the metabolic competencies of such consortia and enable these entities to colonize otherwise inaccessible habitats (Cavanaugh et al., 2006; Kleiner et al., 2012). However, benefits of symbionts for their hosts may vary with ecological conditions or life-stages (Polz et al., 2000; Hay et al., 2004) and are variable in time. In addition, these consortia do not implicitly suggest mutual benefits, but can be sometimes regarded as a trap for both partners, with no chance to escape the relationship. In such a case it will be interesting to study how they can cope as an entity in a new ecological niche and/or compete with uninfected individuals of the host's species (Görtz and Fokin, 2009).

Although most studies on symbioses focus on intracellular microbes, the first step in evolutionary development of eukaryotes may have been the formation of consortia with ectosymbionts offering protection against environmental and physiological hazards (Rosati, 2004). Symbioses, and endosymbioses in particular, drastically accelerate evolutionary changes in genomes of the symbiotic partners, which can be seen as the result of physical proximity and growing liaison of completely different organisms (Shinzato and Kamagata, 2010). Thereby, the establishment of symbiotic interactions typically seems to be established due to specific metabolic capabilities of the symbionts (Hoffmeister and Martin, 2003; Nowack and Melkonian, 2010). In eutrophic, stable environments, however, endosymbioses could be more based on enslavement (meaning parasitism with the ciliate as parasite) than on true mutualistic relationships (see Nowack and Melkonian, 2010).

The incorporation of prokaryotes into symbiotic relationships is assumed to be a selective process, but some ciliates such as *Uronema* sp. are non-selective feeders (Alonso et al., 2000) and thus do not incorporate potential symbionts in a directed manner. Symbionts are well protected from the most important predators of pelagic bacteria when living inside ciliates which probably results in lower mortality rates than of free-living bacteria.

Endosymbioses most likely form in a “mature” stage of ecosystem development, e.g., when the limitation of food threatens reproduction and survival of potential partner organisms. High densities lead to increased intraspecific competition, which favors establishment of new consortia with highly efficient nutrient transfer among symbiotic partners (Nakajima et al., 2009). To facilitate such a transfer of nutrients, the host generates a symbiosome (i.e., a membrane surrounding the endosymbiont) that requires membrane modification to allow transport of otherwise excreted inorganic nutrients or metabolites to and from the symbionts (Yellowlees et al., 2008). The development of these transport systems suggests stable symbioses. On the other hand, it is likely that the host commonly replaces its endosymbionts due to Müller's ratchet—the genetic bottleneck effect that causes genetic depletion of the symbionts (Hackstein et al., 2004; Shinzato and Kamagata, 2010).

Interestingly, geographically separated populations of ciliates may be colonized by different symbiotic genotypes (Fokin et al., 2005; Summerer et al., 2008) and source communities. For example, Bernhard et al. (2000) investigated 15 ciliate species from the Santa Barbara Basin and only found two species without symbionts, ten species with ectosymbionts, five species with endosymbionts, and two species with both. Although the investigated environment is a highly selective one, and thus these findings cannot be easily generalized, it is worth underlining that about one third of the ciliate's body can be occupied by symbionts. This unambiguously demonstrates their potential importance for the host. Ciliates can host bacteria, archaea, and eukaryotes as symbionts. Most of the eukaryotic symbionts are photoautotrophs but there are also a few descriptions of other eukaryotes in ciliates available (e.g., Görtz and Dieckmann, 1987; Fokin et al., 2008). To date, 250 ciliate species are published harboring bacterial symbionts whereby symbiosis seems to be much more likely in nature than in laboratory cultures (see Fokin, 2012). To better understand such interactions, a deeper knowledge of the true diversity of ciliates carrying pro- and eukaryotic symbionts is required, particularly since the majority of aquatic ciliates may support symbiotic microorganisms (Fenchel et al., 1977; Finlay et al., 1996). In addition, the diversity of ciliates may be equalled or even exceeded by that of the symbionts as several ciliates simultaneously support two or more genotypes of symbionts (see Finlay et al., 1996).

## Types of ciliate symbioses

**Figure 1:**
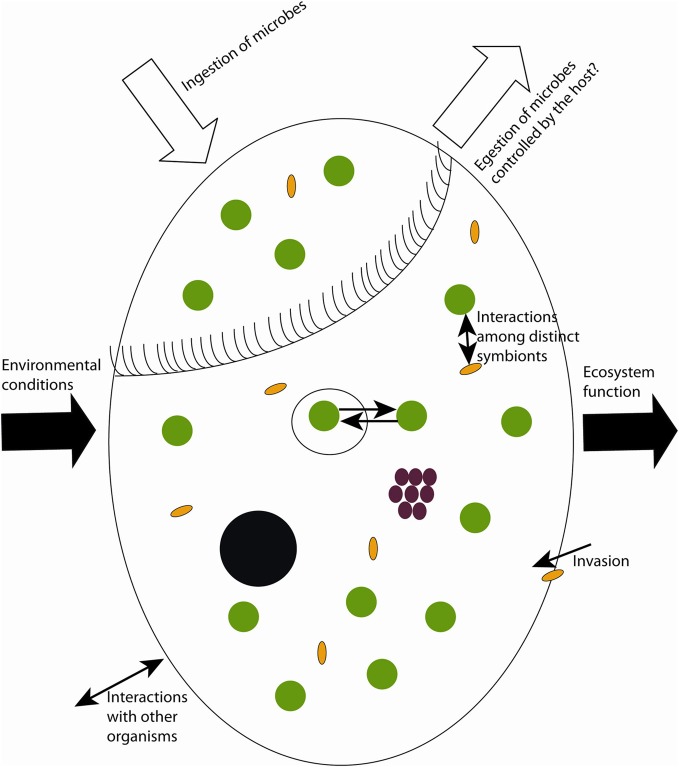
**Sketch of possible dynamics in symbioses with aquatic ciliates as host.** Black circle = macronucleus, white big circle = food vacuoles, green circles = phototrophs, brown circles = chemoautotrophs, yellow ovals = heterotrophic prokaryotes.

To date, research has focused primarily on three major groups of symbionts in ciliates: phototrophic (including pseudo-symbiosis by chloroplast retention), chemosynthetic, and heterotrophic. Studied photoautotrophic groups include zoochlorellae in freshwater ciliates (e.g., *Stentor*, *Paramecium*), dinoflagellates and cryptophytes in marine ciliates (e.g., *Mesodinium*), and kleptochloroplasts in both marine and freshwater ecosystems (e.g., *Strombidium*). Lithoautotrophic symbionts studied include methanogens and sulfur oxidizers in anaerobic or microaerobic ciliates, and heterotrophic symbionts include *Paramecium* and its parasite *Holospora*, *Caedibacter* (the so-called “killer-symbiont”), and *Euplotes* with *Polynucleobacter necessarius*—a stable and inseparable symbiosis. These well-studied interactions appear to be highly specific and to result from long-term co-evolution, suggesting stable and highly conserved systems. Nevertheless, insights into interactions between the single symbiont species and different hosts, and comparison of sequence data from free-living with symbiotic microorganisms indicate that ciliate symbioses represent rather open and highly dynamic entities rather than closed systems (Figure 1).

### Photoautotrophic symbionts

Symbioses of ciliates with phototrophs have arisen independently and repeatedly in freshwater and marine systems (Esteban et al., 2010), resulting in a broad range of relationships. Most symbiotic phototrophs are eukaryotes or chloroplasts and it seems likely that the main function of eukaryotic endosymbionts is photosynthesis (Nowack and Melkonian, 2010). Interactions between the ciliate and its phototrophic symbionts range from brief associations of two facultative partners (e.g., limited retention of photosynthetic prey cells or their organelles) to permanent and even obligate symbioses (Nowack and Melkonian, 2010). Because the primary function of phototrophic symbionts is thought to be the supply of food to the host, such consortia are expected primarily in environments that are otherwise limited in organic food sources. However, mixotrophic ciliate species also commonly occur in eutrophic waters. They often exhibit phototactic behavior (Dolan, 1992), thereby providing their symbionts optimal conditions for photosynthesis. Nearly 25% of aquatic ciliates contain internal chloroplasts or algae (Foissner et al., 1999), making them less dependent on organic food supply from their environment. In addition, harboring a phototrophic endosymbiont may also allow the coexistence of competitors (Müller et al., 2012). These host-symbiont systems persist as obligate and facultative symbioses, whereby both often include a variable number of symbionts depending on season, light, temperature, and other environmental variables. Interestingly, the host seems to be capable of controlling the transfer of various compounds, in particular nitrogen, to the algal symbionts to regulate algal growth (Esteban et al., 2010). A complex cell-to-cell recognition process, in which modifications of algal cell wall structures are crucial, is potentially the basis for initiation and establishment of algal endosymbiosis (Lee et al., 1985).

In marine systems, the presence of kleptochloroplasts dominates besides a number of internal dinoflagellates and cryptophytes. In addition, *Strombidium purpureum* contains a non-sulfur purple bacterium capable of anoxygenic photosynthesis (Fenchel and Bernard, 1993a,b), and *Codonella* sp. is reported as the sole marine ciliate to harbor a cyanobacterium (Esteban et al., 2010). On the other hand, in freshwater systems so-called “zoochlorellae” (unicellular green algae living inside other organisms) predominate. So far, only a single freshwater ciliate species is known to contain the photoautotrophic phytoflagellate *Chlamydomonas* sp. as endosymbiont, whereas others also harbor kleptochloroplasts (Esteban et al., 2010). Sometimes, however, it remains unclear whether the internal compartments originate from endosymbiotic algae or chloroplasts.

#### Kleptochloroplasts

Kleptoplastidy is the ability of a heterotrophic organism to sequester plastids from algae by keeping the chloroplast intact. The plastids are maintained within the host and can be used for photosynthesis. Kleptoplastidy can therefore be defined as predation with farming of the prey organelles (Nowack and Melkonian, 2010). Kleptoplastids may represent an intermediate step in the acquisition of functional plastids or phototrophic symbionts (Nowack and Melkonian, 2010). For example, there are intact chloroplasts in the marine ciliates *Prorodon* and *Strombidium* that are not situated in vacuoles and are not digested by the host (Blackbourn et al., 1973). Dale and Dahl (1987) reported on the presence of symbiotic chloroplasts in *Strombidium*, *Prorodon*, and *Mesodinium rubrum*. Chloroplasts—often called “pseudo-symbionts”—also remain in *Tontonia* and *Laboea*, originating from incorporated prey (Dolan and Pérez, 2000). *Laboea strobila* sequesters photosynthetically functional chloroplasts derived from ingested algae (see Stoecker et al., 1988), and hence these ciliates require both light and algal food for its growth. In addition, the freshwater ciliate *Histiobalantium natans* contains sequestered chloroplasts and mitochondria which enable this aerobic ciliate to even survive and grow under anoxic conditions (Esteban et al., 2009). The duration of chloroplasts actively existing inside ciliates varies with species ranging from hours to a few days (Dolan, 1992) and environmental conditions, particularly light availability.

*Mesodinium rubrum* (= *Myrionecta rubra*) is a particularly interesting case. Although almost completely phototrophic, it is an obligate mixotrophic ciliate. Long included with zooplankton in plankton analyses (Crawford, 1989), it has a high photosynthetic activity. There is ongoing debate whether *M. rubrum* contains chloroplasts or true symbionts, i.e., cryptomonades (see Esteban et al., 2010). What is clear is that they are retained throughout the year (Crawford, 1989) and need only less than one prey algal cell per generation for maximal growth (Hansen and Fenchel, 2006). It is possible that previous studies with *M. rubrum* containing symbionts or chloroplasts have been performed on functionally different clones or even different taxa (Montagnes et al., 2008). The great success of these symbiotic associations is based on the ciliate's motility that enables the otherwise passive planktonic autotrophic partner or chloroplast to optimize its light and nutrient conditions (Wilkerson and Grunseich, 1990).

#### Eukaryotic photoautotrophic endosymbionts

*Strombidium* and *Laboea strobila* often contain endosymbiotic algae as reported for the Baltic Sea (Mironova et al., 2009). This contradicts the observations that these ciliates primarily contain kleptochloroplasts. On the other hand, it seems likely that also here different functional strains were investigated. Another marine ciliate with phototrophic endosymbionts is *Maristentor dinoferus*, which harbors 500–800 symbiotic algal cells—all phylogenetically related to the dinoflagellate *Symbiodinium* (Lobban et al., 2002). Whereas the ciliates reshape at night, the symbionts are spread out in the cap during light, but are mostly moved into the stalk during darkness. These changes, however, are hardly a direct response to light availability (Lobban et al., 2002). Additionally, in *M. dinoferus*, mycosporine-like amino acids are most likely produced by the symbiont and protect the host against UV irradiation (Sommaruga et al., 2006). Therefore, a possible advantage from the endosymbiotic dinoflagellate is the protection of the host's intracellular macromolecules including DNA by shading the endosymbionts when ciliates are exposed to high solar UV irradiation—typically in transparent and oligotrophic waters (Sommaruga et al., 2006).

In freshwater systems, ciliates predominantly contain unicellular green algae, conventionally called zoochlorellae. We will keep this term in the following section. Reisser (1981) postulated for features of symbiotic associations with zoochlorellae: (1) a perialgal vacuole with one vacuole per alga which involves a recognition process between alga and host, (2) growth support of the host by symbiotic algae, and (3) host-symbiont specificity. Interestingly, algal symbionts of freshwater ciliates are of polyphyletic origin (Pröschold et al., 2011) indicating their independent development. Summerer et al. (2008) found a homogenous zoochlorellae group of different ciliate species from one lake, but clearly different zoochlorellae in one of those ciliate species occurring in another lake suggesting repeated incorporation of potential symbionts from the environment. All phototrophic endosymbionts in freshwater systems seem to support the host's growth by maltose excretion in a pH-depending manner (Reisser, 1981). One important factor controlling the endosymbiont's population size is the ciliate's relative need of the autotrophic mode of nutrition which is largely dependent on environmental conditions (Woelfl and Geller, 2002).

There are obligate as well as facultative symbioses with phototrophs: *Platyophrya chlorelligera*, for instance, contains zoochlorella cells at all life stages—even in cysts. On the other hand, euplotid ciliates seem to contain algae only in summer months (Dolan, 1992). To the contrary, for freshwater ciliates the absence or presence of zoochlorellae can be used for species identification as with *Stentor* sp. (Foissner and Wölfl, 1994) and seem thus to be permanent. *Paramecium bursaria* also harbors facultative algal symbionts, which show a membrane-membrane reaction in the host's food vacuole and, thus, prevent digestion (Dolan, 1992). Permanent symbioses in *Paramecium* seem to be restricted to *Chlorella* taxa (Summerer et al., 2007). These algae can provide up to 85% of the photosynthetic fixed carbon to the host (Muscatine et al., 1967), but large numbers of potential algal symbionts are digested (Karakashian and Karakashian, 1973). Non-digested algal endosymbionts are surrounded by a perialgal membrane produced from the host (Meier and Wiessner, 1989) and the endosymbionts cover between 10–56% of the ciliate's volume (Sud, 1968). In lab experiments, *P. bursaria* established stable symbioses with all tested *Chlorella* symbionts originating from various ciliates, but never with symbiotic *Chlorella* of the cnidarian *Hydra* sp. or free-living ones (Summerer et al., 2007). Despite clear preferences for their native *Chlorella*, the host-symbiont relationship in *P. bursaria* is flexible and adapts to environmental changes by accepting non-native *Chlorella* (Summerer et al., 2007). Algal symbionts of all *P. bursaria* strains of different origin could be assigned into one phylogenetic cluster apart from the other ciliate symbionts but split into two distinct lineages on the basis of biogeographic origin (Summerer et al., 2008). Species-specific symbioses were long assumed, but in different *P. bursaria* strains also different algal symbionts exist and at least five different algal species are known for this ciliate species (Pröschold et al., 2011). Summerer et al. (2008) stated that there is no more than one *Chlorella* genotype simultaneously in a single host population but they also cited studies on marine *Symbiodinium* symbioses which have clearly shown that one host can harbor more than one genotype at the same time (Santos et al., 2004). An interesting observation is that *Paramecium* with endosymbiotic zoochlorellae is not preyed as much as without algal symbionts (Berger, 1980). Another function is likely to be photoprotection of symbiotic *Chlorell*a by shading of sensitive cell compartments by a specific arrangement of the algal symbionts, depending on visible light and UV irradiation (Summerer et al., 2009). However, it may also be possible that there is higher grazing pressure on some ciliates containing algae or chloroplasts as they are more visible in the euphotic zone (Dolan, 1992) suggesting adaptations of the ciliates by either symbiosis or behavior.

The abundance of symbionts varies with species, size, and physiological status of the host, but there are at least hundreds of them (Foissner and Wölfl, 1994). As season also influences the host's physiology, it also affects the number of symbionts inside the ciliate host (Beaver and Crisman, 1989).

The ciliate *Climacostomum virens* contains *Chlorella* as symbionts in its cytoplasm and retains them over many generations through cell division and sexual production of the ciliate (Karajan et al., 2007). However, it is possible to grow ciliates as well as algae separately (Karajan et al., 2007). Another freshwater ciliate *Ophrydium naumanni* also contains *Chlorella* as observed in an oligotrophic South Andean lake in Argentina (Queimalinos et al., 1999). These symbionts give the ciliate the ability to effectively exploit the water column in oligotrophic-high-light ecosystems (Queimalinos et al., 1999).

### Lithoautotrophic symbionts

Like phototrophic endosymbionts, lithoautotrophic symbionts can fix carbon and transfer some of their metabolites to the host. These symbionts are mainly found in anaerobic or microaerophilic ciliates. Endosymbiotic methanogens, for example, also occur in symbiotic ciliates of higher organisms and are thus responsible for the production of the green house gas methane, e.g., in cows. Symbiotic methanogenesis in aquatic systems, however, seems to be only of minor importance as phagotrophy under anoxic conditions only leads to slow growth rates (Fenchel and Finlay, 2010). Methanogenic symbionts exist in marine as well as freshwater ciliates and their methanogenic activity depends on the host's metabolism and growth yielding 0.35–7 pmol methane per ciliate and hour at maximal growth rates (Fenchel and Finlay, 1992). Methanogenesis does take place also at low oxygen concentrations in the environment, whereby the most characteristic interaction of methanogens and their host is the syntrophic transfer of hydrogen (Fenchel and Finlay, 2010).

Symbioses with methanogens have been formed repeatedly and independently (e.g., Shinzato and Kamagata, 2010), whereby distantly related ciliate species only contain closely related symbionts and closely related ciliate species only distantly related methanogens (Embley and Finlay, 1993). Despite multiple acquisitions of methanogenic endosymbionts, there is only one unique origin of hydrogenosomes, from mitochondria derived organelles producing hydrogen (Hackstein et al., 2004). There is a close phylogenetic relationship between the endosymbionts and free-living methanogenic archaea arguing for multiple acquisitions from environmental sources but also vertical transmission of endosymbionts has been reported (Van Hoek et al., 2000). The success of symbiosis reconstruction suggests that methanogenic symbiont and host ciliate might recognize each other probably due to membrane structures of the host but not by highly specific means, which could allow for a relatively easy symbiont replacement of anaerobic ciliates (Shinzato and Kamagata, 2010). Today, it is still unclear which factors are involved in the establishment and maintaining of symbiosis in anaerobic protozoa (Shinzato and Kamagata, 2010). It has been also suggested that there is no routine uptake of these endosymbionts but only if renewal of the symbiotic gene pool is needed (Hackstein et al., 2004), which is supported by the fact that strains of *Trimyema compressum* lost their methanogens when kept under cultural conditions (Goosen et al., 1990) without acquiring new ones. The host's benefit of harboring methanogens has been nicely demonstrated in cultures by inhibiting the methanogenic symbionts resulting in a reduction in the host's growth rate by 30% in two of three tested strains (Fenchel and Finlay, 1991a). This observation implies that different developmental stages of symbioses occur in different ciliate species.

*Trimyema* species do not require very specific methanogenic symbionts (Shinzato et al., 2007). The genus *Trimyema* in general contains different methanogenic species with also differences in stability of symbioses (Finlay et al., 1993). *Trimyema compressum* is the only ciliate of this genus known to harbor in addition to the methanogenic archaea some heterotrophic bacteria (Shinzato and Kamagata, 2010).

Narayanan et al. (2009) found *Methanosaeta*, an acetotrophic archaeum, in *Metopus* sp. The methanogenic endosymbionts of *Metopus paleformis* seem to have no effect on the methane concentration in the water (Biagini et al., 1998). On the other hand, *Methanoplanus endosymbiosus* is an endosymbiont in *Metopus contortus* with measurable *in situ* methanogenic activity and grows on hydrogen and carbon dioxide or formate (Van Bruggen et al., 1986). In this symbiosis, a constant number of 5000 hydrogenosomes and 3500 methanogenic symbionts occur, whereby the reproduction of methanogens controls the growth cycle of the host (Fenchel and Finlay, 1991b). *Plagiopyla nasuta* contains the methanogenic *Methanobacterium formicium* which could not be isolated so far (Goosen et al., 1988) and, hence, its specific role for the host is unknown.

The sulfur-oxidizing symbionts, instead, may function to link the sulfur cycle with cycling of carbon and nitrogen (Edgcomb et al., 2011). The host can serve as a shuttle between oxic and anoxic water layers so that the symbionts can use H_2_S or methane as an energy source and oxygen as an electron acceptor whereby also nitrate can be used at least periodically (Cavanaugh et al., 2006). The symbionts have the same generation times as the host and can reach high densities on it (Fenchel and Ramsing, 1992).

In *Zoothamnium niveum* the sulfur-oxidizing bacterium Candidatus *Thiobios zoothamnicoli* was found (Dubilier et al., 2008). Candidatus *T. zoothamnicoli* is only distantly related to previously identified groups of thiotrophic symbionts with highest similarity to a free-living strain (Rinke et al., 2006). Ectosymbionts form a monolayer on *Z. niveum*, which covers the entire colony except for parts of the stalk (Polz et al., 2000). Once in a while, the ciliates rapidly contract and completely immerse themselves in the sulfidic boundary layer. During the subsequent slow expansion they drag sulfidic water into the oxic ambient water (Ott et al., 1998). *Zoothamnium* feeds largely on the symbiotic bacteria detached after contracting (Ott and Bright, 2004) and represents the major food source for the ciliate (Polz et al., 2000). *Zoothamnium niveum* was also found to be almost completely covered by chemolithoautotrophic sulfur-oxidizing bacteria in a mangrove island of the Belize Barrier Reef, which give them a white color (Ott and Bright, 2004).

Only half of the biomass of the mouthless marine interstitial ciliate *Kentrophorus* is indeed ciliate, the remainder is a coat of sulfur-oxidizing bacteria, which is the basis of the ciliate's diet by periodically pushing in its cell surface and digesting the bacteria (Fenchel and Finlay, 1989). These bacteria need both sulfide and oxygen for autotrophic carbon fixation which only coexists in nature in narrow, changing and often unpredictable microzones (Ott and Bright, 2004).

### Organoheterotrophic symbionts

The above mentioned interactions represent mutualistic relationships or interactions which are at least positive for the host whereas heterotrophic symbionts also include transitions to parasites. In general, there might be larger difficulties of integrating a prokaryotic cell into a eukaryotic system than vice versa, mainly due to differences in genome structures and the need of developing a protein transport mechanism to the prokaryotic symbiont (Nowack and Melkonian, 2010). Ciliates may represent genetic “melting pot” promoting cross-species genetic exchange as a result of the co-occurrence of different intracellular bacteria (Nowack and Melkonian, 2010; Lamrabet et al., 2012).

Symbionts providing defense for the host have only been identified for ciliates so far (Gast et al., 2009). Fokin et al. (2005) reported that there are about 200 ciliate species recorded with bacterial symbionts, which is likely to be only a small part of their true number. Most of the marine ciliates investigated by TEM harbor a flora of ecto- and endosymbiotic bacteria (Fenchel et al., 1977). Finlay and Esteban (1998) also stated that most ciliates host endo- or ectosymbiotic organisms, however, they also reported that the diversity and ecological role of these symbionts are hardly investigated. Some of these intracellular bacteria are surrounded by the host's membrane; others not (Görtz and Fokin, 2009). Unfortunately, for the majority of the endosymbiotic bacteria reported in protists, not much more than a morphological description is available, precluding any conclusions about their physiological role as well as a clear recognition of the bacteria as endosymbionts, pathogens, or prey (Nowack and Melkonian, 2010). Intracellular microorganisms typically show low abundance in the host cell (Görtz and Brigge, 1998) and can be found in various cell compartments and in different ciliate species (Görtz, 2001). The symbioses with heterotrophic bacteria appear as highly variable and dynamic with differences on ciliate population levels—and asides the three above mentioned model systems with (1) *Holospora*, (2) *Caedibacter*, and (3) *Polynucleobacter* these interactions are poorly understood and studied.

#### Ciliate symbioses with holospora spp.

The first description of *Holospora* bacteria was already given in 1890 by Hafkine (Fokin et al., 1996). The endonuclear symbiont *Holospora obtusa* is the closest relative to mitochondria known to date (Görtz and Fokin, 2009). There are at least two groups of *Holospora* in *Paramecium* which differ in behavior during host division (Fokin et al., 1996). *Holospora* may be regarded as truly parasitic but has no effect on the host's growth rate under favorable conditions (Görtz and Brigge, 1998). Their metabolic interactions leading to a higher temperature tolerance of *Paramecium caudatum* infected with *Holospora obtusa* is not understood at all (Dohra et al., 1998). Modern functional genomics and proteomics could potentially resolve the ecological role of such interactions.

#### Ciliate symbioses with caedibacter spp.

The transition from mutualism to parasitism can be highly variable as for the killer-symbiont *Caedibacter* spp., which is able to produce a toxin against potential competitors but may also overgrow the host cell (Schmidt et al., 1987). So far, neither the killing toxins nor the mechanism by which paramecia infected with *Caedibacter* resist being killed have been identified (Görtz and Brigge, 1998). It is known, however, that only *Caedibacter* bearing phages or plasmids may confer the killer trait to their host besides in *C. taeniospiralis* a plasmid is responsible for the formation of R-bodies and thus toxin production (Quackenbush and Burbach, 1983; Heruth et al., 1994). Interestingly, when a symbiont-free host cell, which previously harbored killer symbionts, is infected with nonkiller-symbionts, the ciliate proves to be an active killer again. This suggests that extrachromosomal elements (e.g., plasmid) or phages from the killer symbiont are left in the ciliate and can be introduced into the nonkiller bacterium, which in turn is transformed into a killer symbiont (Görtz and Brigge, 1998). Killer symbionts are also found in other ciliates such as *Euplotes*, *Parauronema acutum* (see Görtz and Brigge, 1998), and *Spirostomum ambiguum* (Fokin et al., 2003).

#### Ciliate symbioses with Polynucleobacter necessarius

*Polynucleobacter necessarius* are considered as the most recent known obligate symbionts at the moment (Vannini et al., 2007). The ciliate *Euplotes* needs *Polynucleobacter* for its survival (Heckmann, 1975; Heckmann et al., 1983; Vannini et al., 2005a), whereby *Polynucleobacter* is neither infectious nor pathogenic. Instead, the bacterium works more like an organelle, but its precise function inside the ciliate is still unknown. It has been postulated that it descends from an early symbiont that compensated for a metabolic deficiency of the host (Heckmann et al., 1983) suggesting a stable established symbiosis with a defined physiological function. Recent investigations show that *Polynucleobacter* interferes with the glycogen metabolism of their hosts (Vannini et al., 2007). *Polynucleobacter* is also found in the brackish ciliate *Euplotes harpa* where its removal stops the reproductive cycle of the ciliate (Vannini et al., 2005a). In two of three tested strains of *Euplotes harpa*, *Polynucleobacter* co-occurred with other symbiotic bacteria, whereby the other bacteria showed only low abundances (Vannini et al., 2005a).

#### Ciliate symbioses with other heterotrophic bacteria

The ecological consequences of bacterial symbioses in protozoa are manifold and can even constitute a potential risk for human health when comprising potential pathogenic bacteria (Görtz and Michel, 2003; Ferrantini et al., 2007). Most of the symbionts are not infectious, but the uptake of symbionts can result in a co-infection with infectious microbes (Fokin et al., 2004). Relationships between protists and pathogenic or pathogen-related bacteria including *Legionella*, *Chlamydia*, and *Rickettsiaceae* indicate that there may be potential risks. Protists from sewage plants and composters are frequently infected with microorganisms (Görtz and Maier, 1991; Görtz and Brigge, 1998), e.g., *Rickettsia*-like organisms. However, in the cytoplasm of a marine *Diophrys* species *Rickettsia*-like organisms were also present (Vannini et al., 2003). To be more particular, the ciliate *Diophrys appendiculata* from the Baltic Sea contains specific *Rickettsiaceae*, which possess an independent phylogenetic position within this group (Vannini et al., 2005b). *Euplotes harpa* even contains two *Rickettsia*-like organisms: Candidatus *Anadelfobacter veles* and Candidatus *Cyrtobacter comes* (Vannini et al., 2010). These ciliates represent suitable model systems to study interactions between potentially pathogenic bacteria and their eukaryotic host as well as the resulting ecological consequences (Vannini et al., 2005b). For example, Ogata et al. (2006) postulated that amoeba-like ancestral protists may have served as a genetic “melting pot” where the ancestors of *Rickettsiaceae* and other bacteria promiscuously exchanged genes, eventually leading to their adaptation to the intracellular lifestyle within eukaryotic cells.

Another example for pathogenesis via aquatic ciliates is the fish parasite *Ichthyophthirius multifiliis* containing the following endosymbiotic bacteria: an alphaproteobacterium related to *Rickettsia*, *Sphingobacteria*, and *Flavobacterium columnare*—all with unknown function (Sun et al., 2009). As not all ciliates of this species contain detectable endosymbionts, it is unlikely that endosymbionts play a critical role for the host's physiology. Further, it is unknown whether they play a role for the pathogenic infections by the ciliate or whether they directly affect the immune response of infected fish (Sun et al., 2009). *Francisella noatunensis* is another endosymbiont with potential pathogenic capabilities, which occurs naked and without any other symbiotic genotypes in the marine ciliate *Euplotes raikovi* (Schrallhammer et al., 2011). *Francisella* is a facultative intracellular bacterium, causing severe disease in a broad range of animals including fish. Consequently, aquatic ciliates can serve as reservoirs for pathogenic bacteria with potential severe consequences for animal and human health (Schrallhammer et al., 2011).

The symbiotic relationship between the ciliate *Euplotes magnicirratus* and the bacterium Candidatus *Devosia euplotis* is an example for a permanent and species-specific relationship (Vannini et al., 2004), whereby the bacterium supports the digestion of food organisms (true dependence upon a symbiont, Vannini et al., 2003). Aside of the “classical endosymbiosis,” *Euplotidium* have *Verrucomicrobia*-like ectosymbionts (= **epixenosomes**), which protect their host against the predator *Litonotus lamella* by committing suicide (Rosati et al., 1999). These ciliates keep at least some of their ectosymbionts also in culture (Petroni et al., 2000).

Another type of symbiotic relationships between ciliates and bacteria are rod-shaped **xenosomes**, found in *Parauronema acutum*, which are Gram-negative bacteria comparable in size to *Rickettsia*-like organisms. They exclusively occur in the host cytoplasm and divert together with their host (Soldo, 1987). These xenosomes have multicopies of their genomes (Soldo, 1986). *Parauronema* is infected by direct penetration of the symbiont through the ciliate's cell membranes, and thereafter only a single xenosome is required to establish a permanent infection (Soldo et al., 1993).

Strains of *Trimyema compressum* contain in addition to methanogens also a non-methanogenic prokaryote which can be lost under laboratory culture conditions (Goosen et al., 1990). This suggests that symbioses in *Trimyema* strains are not obligatory and may have a transient character. One of the *Trimyema* bacterial symbionts is only distantly related to other known bacterial species (85% and less) belonging to the *Syntrophomonadaceae* (*Firmicutes*). This suggests that this symbiont is specifically associated to strains of *Trimyema* (Shinzato et al., 2007). Although the absence of the bacterial symbiont after antibiotic treatment considerably decreased the host's growth, the precise role of the bacteria for the ciliate is still unknown (Shinzato et al., 2007). However, it has been supposed that differences in the host's behavior among various strains of *Trimyema* sp. are due to different endosymbiotic communities (Goosen et al., 1990). The presence of multiple symbionts yields a more complex picture of potential symbiotic functions and may even result in a complex functional cycle. For example, in a ciliate closely related to *Parduzcia orbis*, three different types of endosymbionts are present. They are organized within membrane-bound sub-cellular regions and comprise one or two sulfate reducers, a methanogen, and a Type I methanotroph forming synergistic metabolism (Edgcomb et al., 2011).

#### Species-specificity of symbiotic interactions

Based on morphology each ciliate species harbors its specific microbial flora (Fenchel et al., 1977). The marine sediment ciliate *Geleia fossata* can host among 2000–10,000 epibiotic cells. In a few isolates of *Stentor* and *Spirostomum* a number of so far unknown bacterial species have been observed (Görtz, 2001). For *Paramecium* more than 60 intracellular distinct bacteria have been described (Görtz and Fokin, 2009), unfortunately often without depositing the 16S rRNA gene sequence information in public databases. The vent ciliates of the *Folliculinopsis* group harbor multiple phylogenetically distinct symbionts located in different parts of the cell (Kouris, 2009). Symbionts of the ciliate genus *Spirostomum* can be located in the cytoplasm, mitochondria, and macronucleus (Fokin et al., 2003). In general, symbionts in mitochondria are rare and only a minor part of the population is infected as demonstrated for *Halteria geleiana* (Yamataka and Hayashi, 1970) and *Urotricha ovata* (De Puytorac and Grain, 1972). Both *Spirostomum* species investigated seem to permanently maintain their endosymbionts and were—at least partly—colonized by different bacteria (Fokin et al., 2005). The ciliate *Parablepharisma* even shows a specific adaptation for hosting ectosymbionts (Fenchel et al., 1977) leading to a highly host-specific symbiotic bacteria-host relationship. Moreover, the ciliate *Frontonia leucas* hosts an alphaproteobacterial symbiont in its macronucleus (Fokin and Schweikert, 2003). The unequal distribution of two different bacteria in the cytoplasm of *Paramecium* suggests that conditions in various parts of the cytoplasm and other parts of the host cell favor for distinct bacteria and their maintenance in each compartment of the host cell (Fokin et al., 2000). It is likely that the host leads the symbiont to the “right” place with its cytoskeleton (Fokin et al., 2000), which indicate highly specific interactions between the bacterial symbiont and the ciliate host. Different features of the bacteria in regard to their infectivity and their residence place in the host cell can be regarded as a further indication for a highly specific mode of interaction and a great variety of intracellular bacteria (Fokin et al., 2000).

## Current and future approaches to the study of ciliate symbiosis

Studies on symbiotic interactions traditionally include microscopic investigation and phylogenetic characterization of the symbionts using DNA-based approaches. While microscopy (e.g., light microscopy, electron microscopy, confocal laser scanning microscopy) and phylogenetic analysis (e.g., 16S rRNA gene sequencing) allow very detailed structural analysis of host-symbiont interactions they are limited in the study of physiological and functional aspects. As the majority of symbionts do not grow in pure culture most studies today rely on indirect approaches to investigate symbiosis in ciliates (Cavanaugh et al., 2006). Below we provide an overview of current and future technologies that we consider useful in the study of ciliate symbioses.

Symbiotic bacteria can be studied at several levels: morphological (ultra structural), physiological, biochemical as well as on a molecular level (Fokin et al., 2003). The rapid development of new molecular tools greatly improved our understanding of biological mechanisms including organismic interactions (Kitano, 2002; Medina and Sachs, 2010; Hongoh, 2011; Weckwerth, 2011). One major driver of these developments is sequencing technology, which is often key to various studies. Today, next-generation sequencing (NGS) technologies allow high-resolution analysis of single organisms or complex communities on a molecular level at very low costs (Metzker, 2010). More recent developments also put RNA sequencing (transcriptomics) or proteome analysis (proteomics) into focus. The study of mRNA and/or proteins has the advantage that it reveals not only structural information but also indicates genes and metabolic pathways actively expressed by the organisms studied under the conditions the sample was taken (Schneider and Riedel, 2010; Toledo-Arana and Solano, 2010). Using genomics, transcriptomics, and proteomics separately or in combination we can now approach a variety of research questions including: simple phylogenetic profiling of symbiotic communities; comparative genomics of symbiotic microorganisms or hosts; population dynamics of hosts and/or symbionts; or host-symbiont interactions on a molecular level (Kleiner et al., 2012).

While the methods themselves are often routine, sampling and sample preparation became more and more challenging. In particular, working with symbionts can be very difficult due to the close spatial and functional association between hosts and symbionts. For example, ciliates do not only harbor symbiotic bacteria but also graze on them. But how to distinguish between symbiotic bacteria and those contained in food vacuoles? A physical separation of the different fractions is therefore essential—especially when using nucleic acid based approaches (e.g., for symbiotic community profiling) to minimize false interpretations of the data. One option is to label dead cells with propidium monoazide (“live-dead staining”) followed by flow cytometry to separate dead bacteria from living symbionts (Nocker et al., 2007). More recently single-cell approaches became very popular allowing molecular characterization (e.g., genome sequencing) of single bacterial cells (Woyke et al., 2010). For such studies the single cells are usually separated by: (1) micromanipulation using a microscope equipped with a proper micromanipulation device, (2) flow cytometry, or (3) microfluidics (e.g., Hong and Quake, 2003; Brehm-Stecher and Johnson, 2004). Furthermore, density gradient centrifugation can be used to separate bacterial cells from tissues or other organisms (Woyke et al., 2006). Micromanipulation has been successfully used to isolate endosymbiotic bacteria from protists that live in the termite hindgut (Hongoh et al., 2008). The isolated endosymbionts were further characterized by whole genome sequencing revealing that they can fix nitrogen supplying the host with essential nitrogenous compounds.

Working with only a few cells or single cells holds another challenge that needs to be considered: limited nucleic acids and protein concentrations. While culture organisms can be grown to a certain density this is not applicable for single cell approaches. For studies only involving DNA, multiple displacement amplification (MDA) can be used to generate enough material from a few fg of template DNA for subsequent studies (Lasken, 2006). However, there are no standard protocols available yet if working with very little amounts of RNA or proteins for respective transcriptomics or proteomics studies.

Alternatively to working with single-cells, it is also possible to use meta-*omics* approaches (e.g., metagenomics, metatranscriptomics, metaproteomics) to explore host-symbiont interactions (Kleiner et al., 2012). Metagenomics sequencing of bacterial community is a routine application nowadays and can be performed without many difficulties. But it can become problematic when eukaryotic genomes are contained in the samples. While bacterial genomes have sizes ranging from 160 Kbp to 10 Mbp a eukaryotic genome can be as large as 670 Gbp (McGrath and Katz, 2004). So performing metagenome sequencing on a mixture of pro- and eukaryotes can be very challenging and most likely biased toward the eukaryotic host. However, if eukaryotes can be excluded (e.g., by removing the macronucleus of the ciliate host) metagenomics can be a very powerful approach, especially when working with unknown communities. For metatranscriptomics studies, the prokaryote-eukaryote ratio is not as eminent since eukaryotic mRNA has a polyA-tail and thus can be easily separated from the bacterial fraction.

Besides the described sequencing-based approaches there are other methods available that can be used to study organismic interactions. One of these alternatives is microarrays as shown by Barnett et al. (2004). Here a DNA microarray was developed containing probes for the host as well as the symbiont on a single chip allowing to investigating gene expression in both partners simultaneously. However, such microarray studies are only possible if the underlying genomics information of the symbiotic partners is available to design the oligonucleotide probes for the chip. Therefore, microarrays are only of limited use if symbiotic communities are unknown. Genomic or gene sequence information can be also used to design oligonucleotide probes for fluorescence *in situ* hybridization (FISH). In microbial ecology FISH is traditionally being used to label microbes with oligonucleotide probes against the small-subunit ribosomal RNA to distinguish between different phylogenetic lineages (Amann et al., 2001). More recent studies also used FISH to target functional genes by a so-called recognition of individual genes FISH (RING-FISH) (Zwirglmaier et al., 2004; Dziallas et al., 2011). Either approach will be of great use for future studies on symbiotic interactions since they allow to determining how symbionts are structurally and morphologically associated with their hosts and which functions they are able to carry out.

Structural and functional characterization of host-symbiont interactions can be further complemented by additionally collecting metabolic information of the symbiosis. This allows linking genomic and transcriptomic information to real metabolic activities. Metabolomics is a relatively new research field aiming to identify metabolites or intermediates of cellular processes (Macel et al., 2010). The direct study of metabolites has the advantage that it really shows what pathways are active in a cell or community, which cannot be determined by only transcriptomic or proteomic analyses. Another interesting technology in this respect is high-resolution secondary ion mass spectrometry (NanoSIMS) that enables the study of microbial physiology and the use of certain elements (e.g., N, C, S) on a cellular level (e.g., Behrens et al., 2012).

Genomic tools and data available (large-scale sequencing, published genomes, and bioinformatics) could provide increased resolution in the study of bacterial diversity. Standard methods of determining taxon composition using16S rRNA OTUs are known to provide only “coarse” estimates of functional diversity or evolutionarily distinct populations. Improved methods of “species” delineation in bacteria that use DNA sequences will be provided by alternative gene regions (e.g., pseudogenes, intergenic regions; Gomez-Valero et al., 2007) and coalescent-based methods (e.g., Barraclough et al., 2009). Jousselin et al. (2009) used the approach with intergenic (neutrally evolving) regions to delineate fine-scale bacterial “species” and observed a previously unknown host-symbiont co-speciation. Furthermore, genomic information can be also used to develop novel isolation strategies for yet uncultured symbiotic microorganisms as shown previously (Tyson et al., 2005). That would provide the chance of studying “symbioses in action” including the establishment of symbioses of isolates and symbiont-free ciliate hosts.

All these new methods have the potential to provide more detailed information on symbioses and their impact on the respective ecosystem. For example, NanoSIMS may enlighten material flow between different symbionts or symbionts and host and thus give further information on the complex functional interaction within one host and also the dependence of the consortium on different compounds from the environment. Thus, these cutting-edge technologies will not only enlarge our databases, but will help to solve many unanswered ecological questions and also to discover new problems.

## Emerging questions

As is evident from our review, there are many open questions regarding the origin, evolution, maintenance, ecology, and biogeochemical implications of ciliate symbioses. Many of these questions are quite general and their study would provide a better understanding of aquatic ecology, biogeochemistry, and evolution more generally. While several new methods (above) hold promise, we feel that new conceptual approaches are needed to unravel the hidden secrets of bacteria-ciliate interactions. We highlight a number of topics below.

### Do environmental conditions determine the nature of symbioses?

The independent and repeated establishment of different symbioses between aquatic ciliates and microorganisms suggests that environmental conditions at least partly regulate incorporation, maintenance, and termination of these symbioses. Unfortunately, the function of many symbionts is unknown and hence it is still unclear whether there is a directed establishment of symbioses for required functional traits. For example, it seems likely that many aquatic ciliates incorporate and maintain nitrogen-fixing bacteria under nitrogen-limited conditions. If proven to be correct, this would also imply a highly dynamic establishment of symbioses with only loosely linked microbial partners. Alternatively, potentially beneficial symbionts may be retained in the ciliate in low numbers and with reduced activity also when not needed. This might be of particular advantage in unstable and fluctuating environments.

### What are the evolutionary constraints on symbiosis?

Our review shows that ciliate symbiosis has arisen across a broad range of taxa and environments; nonetheless, little is known about the evolutionary basis of the symbiosis. One important question is whether symbionts are species-specific or can affect a range of hosts? If species-specific symbiosis is common, then it may be that host-symbiont co-speciation has given rise to ciliate diversity. If a few small lineages form most symbioses, it suggests only one or a few genomic or genetic mechanisms may be involved, whereas if many unrelated lineages can form symbioses there may be functional redundancy. These and similar questions require broad taxonomic sampling that is increasingly available with large-scale sequencing methods (above) and can employ sequence-based delineation methods (e.g., Jousselin et al., 2009).

### What are the ecological functions of symbionts?

Conventionally, it is thought that stable symbioses are mutualistic relationships in most cases. On the other hand, it is likely that a number of symbioses are established just by chance, e.g., by co-ingestion of infectious symbionts. However, functions of microbial symbionts not only affect the host but can have great implications for global matter cycling, as by nitrogen fixation, phosphorus storage or sulfur transformation, or the ecosystem, as by toxin production, oxygen production in anoxic environments or altered infectivity of pathogenic hosts.

Related to the functionality of symbioses it remains unclear which precise mechanisms and interactions between hosts and symbionts lead to stable and obligate symbioses? Additionally, can neutral symbioses without any measurable effect on both partners be stable? And when symbiosis can be also seen as a trap for both partners: can this symbiosis be stable and how do the partners interact as consortium with their environment including uninfected host organisms?

### Are closed matter cycles formed?

Edgcomb et al. (2011) report on the existence of methanogens and a methanotroph in an individual ciliate suggesting the possibility of closely linked biogeochemical cycles, but even more complex cycles such as for nitrogen seem to be possible. Such complex biogeochemical cycles require multiple symbioses consisting of more than two partners and thus highly specific adaptations and interactions (possibly multitrophic) are necessary. A complex network of symbiotic partners in a single ciliate host is supported by the observation that often more than one symbiont species can be found in a single ciliate.

### Which specific mechanisms allow the host to control the symbiont's physiology?

This question includes not only the need of control mechanisms for metabolic exchange (e.g., nutrient transfer), but also of communication (i.e., signaling). How does the host recognize which symbiont is beneficial at a given time? How can it allocate nutrients to, e.g., a phototrophic symbiont, but not to a symbiont promoting digestion of the host's food? Which mechanisms are involved in the host-symbiont communication?

### Are there different types and mechanisms for symbioses in marine versus freshwater habitats?

Some closely related ciliates occur in marine, brackish, and freshwaters. Thus, for these species different mechanisms in the establishment and maintenance of symbioses are unlikely. However, marine and freshwater ecosystems differ besides salinity in many parameters such as limiting nutrients. This may result in the acquisition of specific microbial functions. For example, most marine systems are limited by nitrogen compounds, thus, acquiring bacteria with the capability of fixing nitrogen is highly advantageous. For these bacteria the host must provide an anoxic or microoxic environment to fix gaseous nitrogen. In freshwater systems on the other hand, phosphate is mainly the limiting factor which may result in symbionts capable of storing phosphate under favorable conditions. Thus, the host might need to move to zones rich in phosphate and establish behavior to circulate this phosphate rich water through its cell.

### Are there different forms of symbioses in anaerobic and aerobic habitats?

If yes, what can we learn for biogeochemical cycling and evolution (e.g., adaptation and speciation of the ciliate and the microbial symbionts)? Containing methanogens seems to be restricted to ciliates living in anoxic or microoxic environments. This symbiosis includes tight coupling between the host's hydrogenosomes and the symbionts, thus even the evolution of hydrogenosomes may represent adaptation to symbiotic lifestyle and a unique niche for symbionts. Another special environment is the bridge between oxic and anoxic conditions where ciliates may favor their symbionts' growth by actively moving between these two zones. On the other hand, ciliates in the oxic zone may provide niches for anaerobic or microaerophilic bacteria in some of their cell compartments.

### Do symbioses increase the speed of microbial gene exchange?

Hereby, it is also interesting if this genetic exchange happens predominately between symbiont and host, symbiont and symbiont or even between symbiont and prey. Additionally the exchange and change of the symbiont's genomic signature after genetic depletion is highly interesting. Which evolutionary principles can be dissected from microbe-ciliate symbioses (e.g., the role of horizontal gene transfer or virus and phages as genetic vectors)?

## Conclusions

**Figure 2:**
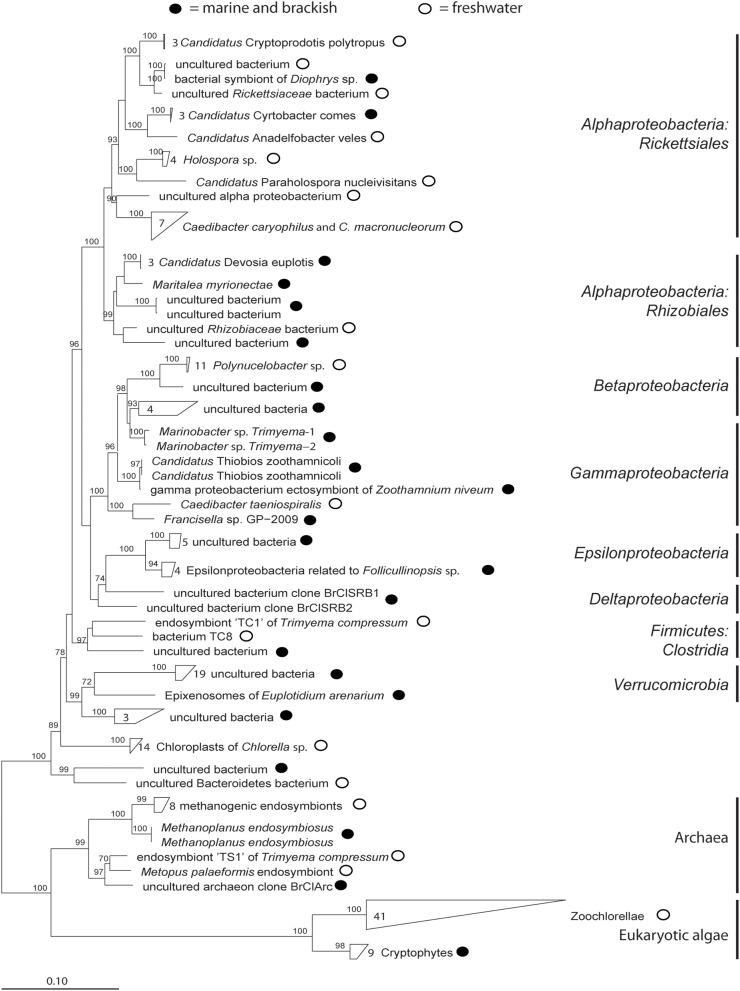
**Phylogenetic tree of published symbionts in aquatic ciliates (accession numbers are given in Table A1).** The tree was calculated with FastTree using the aligned sequences from ARB-SILVA. Bootstrap values are only given ≥70.

Many ciliates contain microorganisms such as algae, Bacteria, and Archaea (Table 3, Figure 2) and interact with these in manydifferent and often unknown ways. As internal algae are easy to recognize using a microscope most studies published to date have been focused on these symbionts—also due to the fact that their interactions with ciliates are mutualistic. Nonetheless, internal bacteria can also comprise commensalistic and parasitic symbionts which might have a great effect on the ecosystem by influencing their host.

**Table 3:** **Overview of ciliates with symbionts**.

	**Examples for ciliate genera**	
	**Marine, brackish**	**Freshwater**
**PHOTOAUTOTROPHIC SYMBIONTS**		
Endosymbionts	*Codonella*	Climacostomum
	*Euplotes*	*Disematostoma*
	*Laboea*	*Euplotes*
	*Maristentor Mesodinium*	*Frontonia*
	*Platyophrya*	*Ophrydium*
	*Strombidium*	*Paramecium*
		*Stentor*
		*Tetrahymena*
Chloroplasts	*Laboea*	*Histiobalantium*
	*Mesodinium*	*Perispira*
	*Prorodon*	
	*Strombidium*	
**LITHOAUTOTROPHIC SYMBIONTS**		
Endosymbiotic methanogens	*Caenomorpha*	*Caenomorpha*
	*Metopus*	*Metopus*
	*Parduzcia*	*Trimyema*
	*Plagiopyla*	
	*Trimyema*	
Ectosymbiotic sulfide oxidizers	*Kentrophorus*	
	*Zoothamnium*	
**ORGANOHETEROTROPHIC SYMBIONTS**		
Mutualistic endosymbionts	*Diophrys*	*Euplotes*
	*Euplotes*	*Paramecium*
	*Parauronema*	
	*Parduzcia*	
Mutualistic ectosymbionts	*Euplotidium*	
		
Parasitic endosymbionts		*Paramecium*
		*Stentor*
Endosymbionts with unknown effects	*Euplotes*	*Frontonia*
	*Folliculinopsis*	*Halteria*
	*Parauronema*	*Ichthyophthirius*
	*Spirostomum*	*Paramecium*
		*Spirostomum*
		*Stentor*
		*Urotricha*
Ectosymbionts with unknown effects	*Geleia*	*Loxophyllum*
	*Paraspathidium*	
	*Tracheloraphis*	

Ecosystem functioning is an important and often little understood parameter in biodiversity research. Unfortunately, symbionts are hardly taken into account although their capability of photosynthesis, sulfur transformation, methanogenesis and their demand on different elements including iron and magnesium is well known. To calculate their importance for regional and global matter cycling will be an interesting and challenging task in upcoming research by also determining their impact for other element cycling such as nitrogen and phosphorus.

Our review shows how diverse and complex symbioses between aquatic ciliates and associated microbes can be and how many gaps in our knowledge still exist. In particular, gaining more information on how symbioses are established and maintained not only extends our scientific knowledge but also may give new insights into species evolution and material cycling and may underline that teamwork can outcompete the individual.

### Conflict of interest statement

The authors declare that the research was conducted in the absence of any commercial or financial relationships that could be construed as a potential conflict of interest.

## Appendix

**Table A1:** **Used published sequences (order according to the phylogenetic tree in Figure 2)**.

**Sequence names**	**Accession numbers**
3 *Candidatus* Cryptoprodotis polytropus	FM201293-5
Uncultured bacterium	AF523878
Bacterial symbiont of *Diophrys* sp.	AJ630204
Uncultured *Rickettsiaceae* bacterium	GQ870455
3 *Candidatus* Cyrtobacter comes	FN552696-8
*Candidatus* Anadelfobacter veles	FN552695
4 *Holospora* sp.	JF713682-3, X58198, AB297813
*Candidatus* Paraholospora nucleivisitans	EU652696
Uncultured alpha proteobacterium	FM201297
7 *Caedibacter caryophilus* and *C. macronucleorum*	AM236090-3, X71837, AJ238683, AY753195
3 *Candidatus* Devosia euplotis	AJ548823-5
*Maritalea myrionectae*	EF988631
Uncultured bacterium	FN999956, FN999980
Uncultured *Rhizobiaceae* bacterium	FM201296
Uncultured bacterium	FN999955
11 *Polynucleobacter* sp.	AJ585515-6, AJ811013-4, AM398080-1, AM397067, CP001010, AM398078, X93019
Uncultured bacterium	FN999982
4 uncultured bacteria	FN999996, FN999962-4
*Marinobacter* sp. *Trimyema*-1, 2	AJ292527, AJ292528
*Candidatus* Thiobios zoothamnicoli	AJ879933, EU439003
Gamma proteobacterium ectosymbiont of *Zoothamnium niveum*	AB544415
*Caedibacter taeniospiralis*	AY102612
*Francisella* sp. GP-2009	FN398155
5 uncultured bacteria	FN999957-60, FN999981
4 Epsilonproteobacteria related to *Follicullinopsis* sp.	GU253370-3
Uncultured bacterium clone BrCISRB1, 2	JF327425, JF327424
Endosymbiont ‘TC1’ of *Trimyema compressum*	AB118592
Bacterium TC8	AB118593
Uncultured bacterium	FN999965
19 uncultured bacteria	FN999947-54, 69-79
Epixenosomes of *Euplotidium arenarium*	Y19169
3 uncultured bacteria	FN999966-67, 46
14 Chloroplasts of *Chlorella* sp.	EF030588-99, 602-3
Uncultured bacterium	FN999968
Uncultured Bacteroidetes bacterium	GQ870456
8 methanogenic endosymbionts	AJ132648-55
*Methanoplanus endosymbiosus*	AB370248, FR733674
Endosymbiont ‘TS1’ of *Trimyema compressum*	AB118591
*Metopus palaeformis* endosymbiont	M86386
Uncultured archeon clone BrCIArc	JF327423
41 Zoochlorellae	EF030554-62, 65-67, FN298917-25, EF044275, EU281549, EF589816, AB206546-50, AB506070-1, AB219527, AY876292, AB191205-7, AB162912-7
9 Cryptophytes	HQ226709, 13, 15, HQ226831, HQ226597, 99, AB4717788-9, DQ452092
